# Changes in Anthropometric Measurements, Body Composition, and Blood Pressure in 6–10-Year-Old Children with Overweight and Obesity in Szczecin During a Year-Long Intervention Program

**DOI:** 10.3390/jcm14103489

**Published:** 2025-05-16

**Authors:** Marta Demiaszkiewicz, Joanna Ratajczak, Dominika Raducha, Tomasz Jackowski, Katarzyna Marcinkiewicz, Ewa Berus, Mieczysław Walczak, Elżbieta Petriczko

**Affiliations:** 1 Department of Allergology, Pomeranian Medical University, 70-111 Szczecin, Poland; marta.demiaszkiewicz@pum.edu.pl; 2 Institute of Physical Culture Sciences, University of Szczecin, 71-065 Szczecin, Poland; joanna.ratajczak@usz.edu.pl; 3 Department of Pediatrics, Endocrinology, Diabetology, Metabolic Disorders and Cardiology of Developmental Age, Pomeranian Medical University, 71-252 Szczecin, Poland; dominika.raducha@pum.edu.pl (D.R.); tomasz.jackowski@pum.edu.pl (T.J.); ewa.berus@pum.edu.pl (E.B.); klchrdz2@pum.edu.pl (M.W.); elzbieta.petriczko@pum.edu.pl (E.P.)

**Keywords:** childhood obesity, body composition, blood pressure, intervention program

## Abstract

**Background:** The growing problem of excess body weight in children is currently one of the most important public health threats. **Methods:** The aim of this study was to assess the impact of a year-long intervention on the body composition and blood pressure of 6–10-year-old children with excess body weight in the city of Szczecin. This study was divided into two parts: stage I was a cross-sectional population-based study (screening in schools) while stage II was an interventional study with before/after comparison. **Results:** A total of 4890 children participated in the first stage, while 515 children qualified for the second stage. During each visit in the intervention stage, anthropometric measurement (height, body weight), body composition analysis using bioelectrical impedance (body fat mass in kilograms, percentage of body fat, muscle mass in kilograms, fat-free mass), and blood pressure measurement were performed. This study did not record a significant effect of the year-long intervention on changes in the blood pressure percentiles of the children. However, there was a significant increase in the number of children with a normal percentage of body fat (92.64% vs. 96.92%) and a significant increase in muscle mass in the majority of children (96.30%, *p* < 0.0001). The body fat percentage, body fat mass, and muscle mass measurements differed between children who were overweight and those who were obese. The average percentage (girls 27.80 ± 2.72 vs. 24.61 ± 3.04; *p* < 0.0001, boys 20.93 ± 4.51 vs. 16.02 ± 1.83; *p* < 0.0001) and mass of body fat (girls 12.12 ± 2.82 vs. 9.44 ± 1.46; *p* = 0.0001, boys 9.73 ± 3.72 vs. 6.16 ± 1.31; *p* = 0.0001), as well as the average muscle mass (31.01 ± 4.6 vs. 26.8 ± 4.08; *p* < 0.0001), were higher in children with obesity compared to those with overweight. **Conclusions:** Further research is necessary to evaluate the effectiveness of therapeutic programs focused on treating excess body weight in children.

## 1. Introduction

The issue of obesity is currently escalating in many countries and is considered one of the most significant threats to public health. Unfortunately, excess body weight affects not only adults but also children. Overweight and obesity are now the most common physical development disorders in children and adolescents. According to data from the World Health Organization (WHO) in 2022, excess body weight was diagnosed in 390 million children worldwide [1]. It is particularly concerning that the average age of children affected by excess body weight is decreasing. However, in some highly developed countries, a plateau or even a decrease in childhood obesity rates can be observed, which may be associated with the promotion of public health promotion obesity prevention programs [2].

The majority of childhood obesity cases are classified as simple obesity, which is linked to a positive energy balance between the amount of energy consumed from food and the energy expended during physical activity. The complications of childhood obesity can affect various organs and systems, and their severity depends on the degree of obesity. It has been proven that excess body fat leads to insulin resistance, glucose intolerance, type 2 diabetes, dyslipidemia [3,4,5], and metabolic-associated fatty liver disease (MAFLD) [6]. Additionally, childhood obesity contributes to obstructive sleep apnea, osteoarthritis, polycystic ovary syndrome, precocious puberty, and fertility problems [7]. One of the most common complications of childhood obesity is arterial hypertension. Although secondary causes of hypertension dominate in the developmental age, primary hypertension has become more prevalent among older children due to the growing number of children with obesity [8]. Many studies indicate a correlation between elevated blood pressure in children and an increased body mass index (BMI), particularly visceral obesity [2,9]. Research shows that, even in individuals with a normal body weight, excess body fat can lead to increased blood pressure and elevated cardiometabolic risk due to associated hyperinsulinemia, insulin resistance, and dyslipidemia [10].

In recent years, bioelectrical impedance analysis (BIA) has become a common and accessible method for assessing the body composition of individuals, particularly their composition of fat tissue. This method is recognized as reliable, repeatable, safe, non-invasive, and relatively inexpensive, which has led to its growing use in clinical practice. Based on the differences in electrical resistance between different fat-free tissues, the device estimates the fat mass, fat-free mass, percentage of body fat, muscle mass, and total body water of an individual. Studies show that body fat measurement using BIA provides reliable results in individuals who are both healthy and obese [11,12], as well as in children [13,14]. BIA allows for the assessment of whether a change in body weight is associated with a reduction in fat mass or, for example, a loss of fat-free mass. BIA results (especially graphical outputs) can be helpful in health education and in strengthening patients’ motivation.

The recommended first-line treatment for childhood obesity is multidisciplinary care aimed at lifestyle modification. It has been proven that the early years of life are crucial in developing food preferences and dietary habits. Therefore, it is important that programs that support lifestyle modification are directed towards younger children and their families.

## 2. Materials and Methods

### 2.1. Study Design

This study consisted of two stages. Stage I was a cross-sectional population-based study. The second stage was an interventional study with before/after comparison. The population that was studied consisted of children aged 6–10 years who were participating in the “The Brave Eight—A Program to Prevent Overweight and Obesity Among 8-Year-Old Children Attending Primary Schools in Szczecin”. The analysed data came from studies conducted between 18 September 2016, and 31 December 2018. The “Brave Eight Program” was fully funded by the City of Szczecin and was implemented after a license was obtained from the Polish Society for Health Programs in Gdańsk [15,16]. This study received a positive opinion from the Bioethics Committee at Pomeranian Medical University in Szczecin (KB-0012/85/15), dated 22 June 2015.

Stage I: This stage involved screening examinations for children in the second and third grades of primary schools in Szczecin. The only exclusion criterion in Stage I was lack of guardian consent to participate in this study. The children underwent anthropometric measurement (height, body weight), blood pressure measurement, body composition analysis using the BIA method, and a physical fitness test (step-test). This stage was conducted by trained nurses from the Non-Public Health Care Facility for School Medicine “SZKOLMED” in Szczecin. The children’s BMI was calculated using the formula body weight [kg]/height [m^2^]. Children with a BMI ≥ 90th percentile according to Polish population standards based on OLAF percentile grids (name of a nationwide, nationally representative epidemiological study (PL0080) aimed at assessing anthropometric parameters) were qualified for the second stage of the program [17].

Stage II: This stage was carried out at the outpatient clinic of the Independent Public Clinical Hospital No. 1 at the Pomeranian Medical University in Szczecin. It consisted of a year-long specialist intervention during which children, along with their parents, attended 4 sessions (in the 1st, 3rd, 6th, and 12th months of the program). During each visit, the children and their parents had consultations with a team of specialists including a doctor, a dietician, a physical activity specialist, and a psychologist, resulting in a total of 16 consultations. During each visit, the following was carried out:

(1) In the doctor’s office, a medical history was taken, a physical examination was performed, and anthropometric measurement (height, body weight), blood pressure measurement, and body composition analysis using the BIA method were conducted. Depending on the information obtained from the medical questionnaire—an integral part of the study—covering sleep hygiene, screen time, exposure to tobacco smoke, existing chronic conditions, current medications, and family medical history, and based on the physical examination, each participant received recommendations related to lifestyle changes and further medical follow-up [18];
(2) In the dietician’s office, children and their parents were given dietary consultations, during which the child’s daily diet was analysed, and individualized recommendations for healthy eating, which were consistent with the study protocol, were provided (“Nutritional recommendation”, Supplementary Materials);
(3) In the physical activity office, fitness tests were performed, such as evaluation of the abdominal and shoulder girdle muscle strength, long jump from a standing position, flexibility assessment, and physical fitness assessment using the step-test. In addition, a questionnaire on the child’s physical activity (participation in physical education classes, extracurricular sports, active leisure time) was conducted, and individual recommendations on physical activity, lifestyle, and screen time reduction were provided [18];
(4) In the psychologist’s office, interviews were conducted that focused on social and family functioning, eating disorders, and stress management. Motivational discussions were held, and goals and methods for achieving them in relation to lifestyle changes were discussed.

Additionally, once a week, throughout the entire duration of the program, educational workshops and sporting events (e.g., cycling trips, swimming classes, scavenger hunts, soccer games) were organized for interested children and their families by the team. A series of educational workshops for teachers and school cafeteria staff was also conducted. Information about the planned activities was available on the website dedicated to “The Brave Eight” and on Facebook.

### 2.2. Sample Size

**Figure 1:**
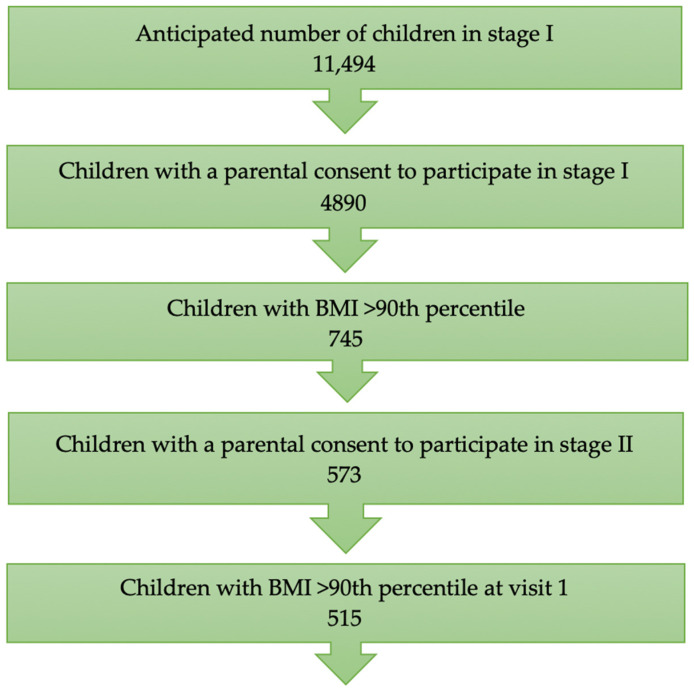
Recruitment process for subsequent stages of this study.

Parental consent for participation in the screening phase of the program (school examinations) was obtained for 4890 children (Figure 1). Excess body weight was diagnosed in 22.6% of the children (1109), including 16.9% with overweight (806) and 6.4% with obesity (303). Due to the public nature of the program, all children attending the second and third grades were examined, resulting in a small number of children who were younger or older than expected. A total of 745 prepubertal children (15.2%) aged 6–10 years with a BMI ≥ 90th percentile by sex and age according to OLAF standards were included in this study [17]. The criteria for study inclusion included obtaining written parental/guardian consent for the child’s participation, age 6–10 years, and a BMI at or above the 90th percentile for sex and age according to OLAF percentile grids [17]. Children whose parents did not consent to the second phase of the program were excluded, and a total of 573 children participated in the subsequent phase. Ultimately, data from 515 children were analysed, as those whose BMI had decreased between the school examination in the first stage and the first visit in the second stage of the program were excluded.

### 2.3. Research Methods

Anthropometric measurement, blood pressure measurement, and body composition analysis were performed in both the first and second stages of the program as follows:

(1) Height was measured using a Harpenden stadiometer, accurate to 1 mm, in a standing, upright position without shoes;
(2) Body weight was measured using a Jawon electronic scale, accurate to 0.1 kg, while the child was in their underwear;
(3) Blood pressure was measured three times using an Omron 2 oscillometric device (OMRON Healthcare Co., Ltd., Kyoto, Japan) with appropriately selected cuffs. The measurements were taken in a sitting position after a few minutes of rest. The average systolic and diastolic blood pressure was calculated from the three readings;
(4) Body composition analysis using bioelectrical impedance analysis (BIA) was performed, in the first stage, using a Jawon Medical X-Contact 350 analyser (SELVAS Healthcare Inc., Daejeon, Republic of Korea) and, in the second stage, a Jawon Medical IOI 353 analyser (SELVAS Healthcare Inc., Daejeon, Republic of Korea). The parameters measured included body fat mass in kilograms, percentage of body fat, muscle mass in kilograms, fat-free mass, and water content.

The reference system was based on Polish percentile grids from the OLAF project for height, body weight, BMI, and blood pressure [17]. For statistical analysis, BMI norms according to the CDC [19] were adopted:

- Normal: BMI < 85th percentile *;
- Overweight: BMI ≥ 85–95th percentile *;
- Obesity: BMI ≥ 95th percentile *.

* for sex and age

Blood pressure norms were adopted according to the recommendations of the Polish Society of Pediatric Nephrology (PTNFD) [20]:

- Normal blood pressure: <90th percentile *;
- High normal blood pressure: ≥90–95th percentile *;
- Hypertension: ≥95th percentile *;

* for sex and age according to the Polish percentile grids from the OLAF project [21].

Since Polish norms regarding body fat content in children are not available, percentage body fat norms for sex and age were adopted based on a German study involving over 22,000 children aged 3–16 years [22]. Unfortunately, there are no established norms for other body composition parameters (muscle mass, fat mass) in children.

### 2.4. Statistical Analysis Methods

The *Z*-score for body weight, height, and BMI was calculated based on the 2000 CDC data available on the CDC website [23]. The LMS parameters were used: median (*M*), generalized coefficient of variation (*S*), and power in the Box–Cox transformation (*L*). The *z*-score was calculated using the following formula:

$$Z=\frac{{\left(\frac{X}{M}\right)}^{L}-1}{L\times S},L\neq 0$$

Statistical analysis was conducted using the STATA 11 statistical software. All tests were considered statistically significant with *p* < 0.05. A significance level of *p* = 0.051–0.099 was considered a trend approaching statistical significance. The Kolmogorov–Smirnov test was used to assess the normality of the distribution of the studied variables. Almost all variables showed normal distribution, and if normality was not met, there was minimal skewness (D_max_) and a large sample size (N). Therefore, Student’s *t*-tests or ANOVA were applied. Group characteristics were described using means, standard deviations, and minimum and maximum values. Pearson’s correlation was used to assess correlations between continuous and nominal variables, with the results being presented as a correlation coefficient (r) and *p*-value. A strong correlation was defined as r > 0.5, and a weak correlation as r > 0.3 but < 0.5. No correction for multiple comparisons was applied due to the exploratory nature of this study.

## 3. Results

### 3.1. Changes in BMI

A statistically significant decrease in the overall BMI percentile (−1.74) and BMI z-score (−0.16) was observed during the year-long program, as shown in Table 1. The greatest reduction in the BMI percentile and z-score occurred between the first and second visits (−0.83 and −0.09, respectively). Nearly 80% of children achieved a reduction in their BMI percentile and z-score during the program (Table 2).

### 3.2. Changes in Blood Pressure

**Figure 2:**
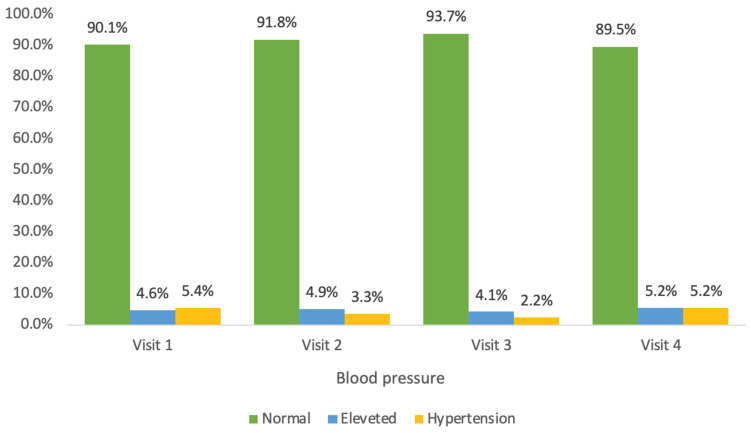
Systolic blood pressure across visits (1–4).

**Figure 3:**
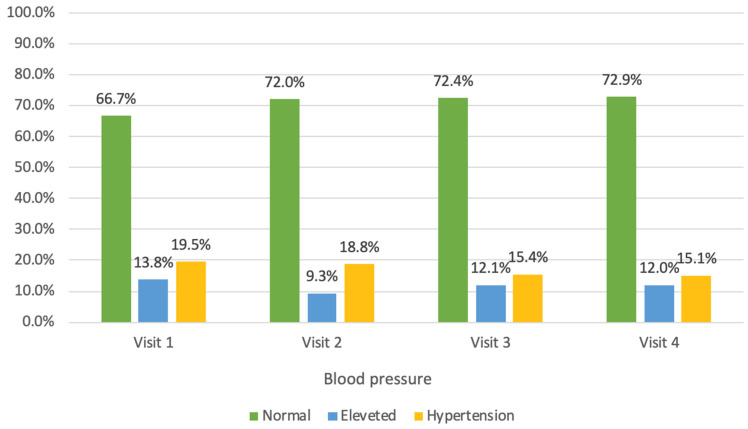
Diastolic blood pressure across visits (1–4).

Normal systolic blood pressure was recorded in 90% of the children during both the first and fourth visits (Figure 2). Elevated systolic blood pressure was diagnosed in 10% of the children, with hypertension being detected in 5.4% of the children. Normal diastolic blood pressure was observed in 66.7% of the children during their first visit, while 33.3% of the children had elevated diastolic blood pressure, including 19.5% with hypertension, as shown in Figure 3. By the fourth visit, normal diastolic blood pressure was recorded in 72.9% of the children, and elevated diastolic blood pressure was recorded in 27.1%, with hypertension being present in 15.1%.

During the year-long program, there was a statistically borderline increase of 3.57 in the mean systolic blood pressure percentile, while a non-significant decrease was observed in the mean diastolic blood pressure percentile (Table 3). The mean systolic blood pressure percentile decreased in 41.5% of the children during the year-long program. The mean diastolic blood pressure percentile decreased in 49.5% of the children.

### 3.3. Changes in Body Composition

#### 3.3.1. Changes in Body Fat Percentage

**Figure 4:**
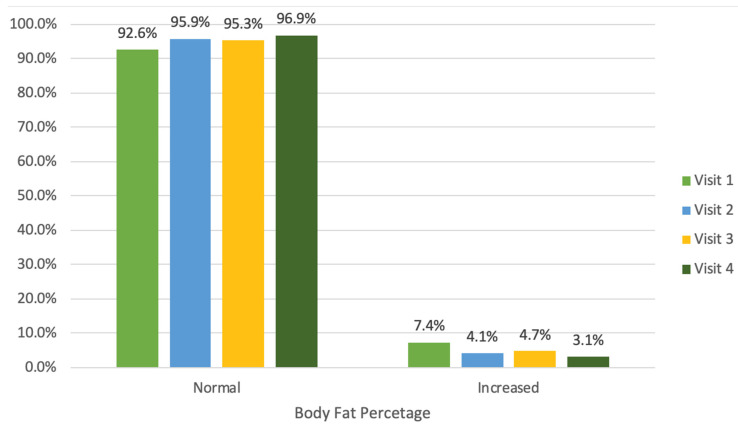
Percentage of children with normal body fat percentage across visits (1–4).

During the year-long program, there was a statistically significant increase in the number of children with a normal body fat percentage (Figure 4). At the first visit, 92.6% of the children had a body fat percentage within the normal range (<90th percentile), which increased to 96.9% by the fourth visit.

A statistically significant decrease in the mean body fat percentage was observed between the first and second visits (−0.5%), as shown in Table 4. Between the first and fourth visits, there was a borderline statistically significant increase of 0.4% in the mean body fat percentage.

#### 3.3.2. Changes in Body Fat Mass

A statistically significant increase of 1.5 kg in the mean body fat mass was observed during the year-long program (Table 5). A reduction in body fat mass was recorded in 17% of the children (Table 6). The largest number of children who achieved a decrease in their body fat mass was observed between the first and second visits (53%).

#### 3.3.3. Changes in Muscle Mass

An increase of 3.4 kg in the mean muscle mass was recorded during the year-long program (Table 7). A total of 96.3% of the children increased their muscle mass (Table 8).

### 3.4. Changes in Parameters in Boys and Girls

The mean BMI percentile and z-score were higher in the boys than in the girls during each visit. A larger decrease in BMI percentile (statistically significant) and BMI z-score (not statistically significant) was observed in the girls compared to the boys during the year-long program. A higher percentage of children with obesity among the boys than among the girls was noted during each visit, as shown in Table 9.

#### 3.4.1. Changes in Blood Pressure Between Boys and Girls

The mean systolic and diastolic blood pressure percentiles were higher in the boys than in the girls during each visit. However, the differences in the changes in systolic and diastolic blood pressure percentiles between the boys and girls throughout the year-long program were not statistically significant. A significant increase in the proportion of girls with normal systolic blood pressure, from 91% to 96%, was observed. This trend was not observed in the boys. A statistically significant increase was noted in the proportion of both girls and boys with normal diastolic blood pressure: from 68% to 72% in the girls, and from 65% to 74% in the boys. Detailed data are presented in Table 10.

#### 3.4.2. Changes in Body Fat Percentage Between Boys and Girls

Initially, a higher proportion of girls (10%) than boys (5%) had elevated body fat percentages. However, by the end of the year-long program, 98% of the girls had achieved body fat percentages within the normal range, while no significant change was observed in the boys (Table 11). During the intervention, 57% of the girls and only 30% of the boys experienced a decrease in their body fat percentage. The mean body fat percentage decreased in the girls during the program, whereas it increased in the boys.

### 3.5. Changes in Parameters in Children with Overweight and Obesity

The mean systolic and diastolic blood pressure percentiles were higher in children with obesity compared to those with overweight, as presented in Table 12.

Both the mean body fat percentage and body fat mass (Table 13 and Table 14), as well as the muscle mass (Table 15), were higher in the children with obesity compared to those with overweight. Initially, a significantly greater reduction in body fat percentage was observed in the children with obesity compared to those who were overweight, but this trend did not persist during subsequent visits.

### 3.6. Pearson Correlations

A strong relationship was demonstrated between the percentage of body fat and anthropometric parameters such as body weight, body weight z-score, BMI, BMI percentile, and BMI z-score, as presented in Table 16.

A strong relationship was also shown between fat mass and anthropometric parameters such as body weight, body weight z-score, height, BMI, BMI percentile, and BMI z-score (Table 16).

Additionally, a strong relationship was demonstrated between muscle mass and anthropometric parameters such as body weight, body weight z-score, height, height z-score, and BMI (Table 16).

The obtained correlations between body composition parameters and blood pressure included only a weak positive correlation between the percentage and mass of body fat and blood pressure and the blood pressure percentile in the boys (Table 17). A weak correlation was also found between both muscle mass and systolic blood pressure and the systolic blood pressure percentile.

A weak correlation was found between an elevated systolic blood pressure and body weight, BMI, fat mass, and muscle mass. No correlation was found between an elevated diastolic blood pressure and the studied parameters.

A strong correlation was demonstrated between a high percentage of body fat and BMI, and a weak correlation was observed between a high percentage of body fat and body weight, body weight z-score, BMI percentile, and BMI z-score.

## 4. Discussion

Although our team visited each school to inform parents about the program and its benefits, relatively few parents were interested in participating. A total of 4890 parents consented to their children participating in the program, which represented about 42% of the guardians. Programs focused on preventing and treating childhood overweight and obesity often face low to moderate parental engagement. Studies indicate that parents initiate participation in weight management programs out of concern for their children’s psychological well-being and health. However, despite their initial commitment, many parents withdraw from the program during the early stages [24]. A low rate of parental consent for participation in this study may result from insufficient awareness of the seriousness of childhood overweight and its broad health and social consequences. Some caregivers who believe their children have a normal body weight do not see the need to verify their assessment using objective methods and, therefore, will not be interested in participating even in the first stage of the program. Fear or shame related to judgment and potential stigmatization of the child by other parents and peers is also a significant factor.

In the screening phase of our study, we diagnosed excess body weight in 23% of the children, including 16.9% who were overweight and 6.4% who were obese. Similar results were obtained in national population studies. In the OLAF study conducted between 2007 and 2012, excess body weight was diagnosed in 21.4% of 8-year-old boys and 17.9% of girls [25]. In the COSI study conducted in 2015–2016, overweight was diagnosed in 23.2% of children aged 6–9 years (using IOTF criteria), with rates of 22.6% in boys and 23.7% in girls [26]. Children who had reduced their BMI to values within the normal range by the time of the first visit in the second stage of the program were excluded from the intervention phase. This improvement was observed in 58 children, who achieved a normal BMI by the time of the first visit. This was one of the desired effects of our program. Conducting measurements at school and informing guardians about their child’s excess body weight, combined with simple recommendations for a healthy diet and increased physical activity, resulted in 10% of the children achieving a normal BMI by the time of their specialist visit (about 3–4 weeks).

Children who achieved the desired effect of a normal BMI (<90th percentile for age and sex) during the year-long program completed their participation in the project. Some children reached a normal BMI by the second or third visit. All children who achieved a normal BMI (the program’s best outcome) were not included in the final analysis of the results which took place after the fourth visit, which marked the end of the program. It is worth considering continued monitoring of this group of children in the future to assess the stability of the achieved outcomes.

The decreasing number of children participating in the program at each stage was due either to children achieving a normal BMI or, more commonly, due to them dropping out. Previous studies have shown varying dropout rates in childhood obesity treatment programs. Reviews of meta-analyses report dropout rates ranging from 25 to 50% or from 3 to 48% [27,28].

Although more children diagnosed in the screening phase had overweight rather than obesity, most of the children who participated in the intervention phase were classified as obese (76%). This may be because parents of children with obesity are more likely to perceive the problem as significant and thus decide to participate in the intervention program. However, according to a meta-analysis by Kelleher, their child’s BMI alone does not necessarily influence parents’ willingness to participate in an intervention program [24]. It is also worth considering whether organizing the visits during the second phase of this study in a university hospital was an encouraging factor or, conversely, a deterrent to program participation. On one hand, such a facility emphasizes the seriousness of the problem and its health consequences; on the other hand, it may discourage some caregivers and children who perceive it as excessive medicalization. It also seems that implementing the intervention at an even earlier age [29] and maintaining it over a longer period could more effectively help consolidate changes in eating habits and increase the daily physical activity of children who participate. In the future, it may be worth considering the inclusion in similar programs of not only children with overweight and obesity but also those at risk of developing overweight. However, attention should be paid to the challenges encountered in maintaining participation among children who reduced their BMI to below the 90th percentile at different stages. A large proportion of them dropped out of the program despite having the opportunity to continue. This indicates a low perceived threat, which may be related to poor health awareness concerning the complications of excessive body weight, including overweight. This highlights how much effort is still needed to promote this knowledge. A significant factor influencing continued participation in similar programs is often the lack of immediate results in terms of weight reduction. Despite discussions about expected outcomes and the pace of changes during the initial medical visits, this element is often decisive in the decision to discontinue participation. It is worth considering ways to increase the attractiveness of activities that promote physical activity and introduce healthy eating habits into everyday life. Given the organizational challenges faced daily by families with children, diversifying the available activities may help maintain engagement from both children and caregivers in the program.

During each visit, a desired reduction in both the mean BMI percentile and BMI z-score was achieved. The greatest reduction occurred between the first and second visits. Throughout the program, the number of children who experienced a decrease in their BMI percentile and z-score increased. Nearly 80% of the children showed a reduction in their BMI percentile and z-score during the year-long intervention. The initial mean BMI percentile was 97, and the z-score BMI was 1.9. After the year-long intervention, these values decreased to 95 and 1.7, respectively. In the meta-analysis by Ells, a slightly higher initial mean BMI z-score (2.25) was found in similar intervention programs for children with excess body weight, with a smaller average reduction in BMI z-score (0.05) [30].

During each visit, the BMI percentile and z-score were higher in the boys than in the girls. Additionally, a higher percentage of boys were classified as obese compared to the girls at every visit. Notably, the decrease in the BMI percentile and z-score was greater in the girls than in the boys throughout the program, suggesting that the girls achieved better outcomes in reducing excess body weight in our program.

Previous randomized clinical trials have reported comparable percentages of children achieving a decrease in their BMI z-score during multidisciplinary interventions. In Reinehr’s study, a 12-month intervention resulted in a BMI z-score reduction in 72% of children [31]. In another study by Reinehr, a reduction in BMI z-score was observed in 77% of children, and interestingly, the reduction was sustained in 66% of children three years later [32]. In Mayerhofer’s study, a 5-month intervention resulted in a BMI z-score reduction in 79% of children, with similar effects observed in both boys and girls [33].

A review of meta-analyses evaluating the impact of multidisciplinary interventions on children with excess body weight shows a reduction in BMI, but the results are not entirely consistent. In Hamid’s study, the effects of multidisciplinary interventions on children aged from 6 to 12 years with overweight and obesity were assessed. Four out of seven studies reported a significant reduction in the BMI z-score in the intervention group compared to the control group, while two studies found no significant differences between the groups [34]. A meta-analysis of 21 studies evaluating changes in BMI and BMI z-score during interventions found moderate benefits in the study group compared to the control group across all studies [35]. A meta-analysis of 14 studies comparing the effects of multidisciplinary interventions to a control group without intervention found improvements in BMI z-score, BMI, and body weight in the intervention group, both after the intervention and during follow-up several months later [36]. Ells’ meta-analysis showed a positive effect of interventions on the BMI z-score of participants [30]. Zolotarjova’s study, which evaluated the effects of multidisciplinary interventions on children aged 4–18 years with severe obesity, reported a reduction in the BMI and BMI z-score of participants in each study. However, despite the favourable impact on cardiovascular risk factors, weight loss was often not sustained. Younger children and boys showed better maintenance of weight loss [37].

Differences in the results of meta-analyses may stem from the variety of interventions that are applied. Clinical studies vary significantly in terms of the interventions that are undertaken. Some studies involved isolated interventions, while others were multidisciplinary. The programs were conducted in different settings—at school, at home, in healthcare facilities, or a combination of these. The duration of interventions, frequency of visits, and intensity of physical activities also varied. Some studies merely provided guidance on recommended physical activity, while others included supervised exercise. The level of parental involvement in the intervention process also varied. This diversity in study designs makes it difficult to compare their results.

In our study, an alarmingly high percentage of children had abnormal blood pressure. Elevated systolic blood pressure was diagnosed in 10% of children, with hypertension detected in 5%. Nearly one-third of the children were found to have elevated diastolic blood pressure, with hypertension being diagnosed in 20%. During the year-long program, the percentage of children with normal diastolic blood pressure increased to 73%. However, no significant changes were observed for systolic blood pressure.

During the year-long intervention, a significant increase in the proportion of girls with normal systolic blood pressure was observed (from 92% to 96%). No such change was seen in the boys. A significant increase in the percentage of children with normal diastolic blood pressure was observed in both the girls (from 68% to 72%) and boys (from 65% to 74%).

The mean blood pressure, as well as the systolic and diastolic percentiles, were higher in the children with obesity than in those with overweight. Previous studies confirm that elevated systolic and diastolic blood pressure is more common in children with overweight and obesity compared to healthy children [9,10,31,38,39,40].

A review of previous meta-analyses indicates a positive effect of interventions on blood pressure values in children with excess body weight. Cai’s meta-analysis demonstrated a moderate impact of lifestyle change interventions on the blood pressure of children with excess body weight. Programs that focused solely on physical activity or dietary interventions did not show a significant effect of lowering blood pressure. However, interdisciplinary interventions significantly reduced the blood pressure of participants [41,42]. In Rajjo’s study, multidisciplinary interventions combining dietary education, physical activity, and behavioural therapy resulted in a significant reduction in both BMI and systolic and diastolic blood pressure. Most of the programs also observed improvements in body composition. Interestingly, in contrast to previously mentioned studies, interventions focused only on physical activity also led to a reduction in systolic blood pressure [43]. Ho’s study found that short-term interventions lasting up to 6 months resulted in a decrease in diastolic blood pressure, while studies lasting more than a year showed a reduction in systolic blood pressure. The best results in regard to improving the blood pressure of children who are obese were achieved in an intensive, 12-week school program that included vigorous physical exercise and dietary counselling [44]. Peirson’s study showed that even a moderate reduction in BMI in intervention programs had a significant effect on reducing the blood pressure in children with excess body weight [35]. A significant decrease in blood pressure was observed in children with a BMI z-score reduction as small as 0.1 [30]. A meta-analysis of interdisciplinary interventions in children with excess body weight demonstrated that a decrease in BMI during a program was associated with a reduction in blood pressure. In children who did not achieve a BMI reduction, there was no decrease in blood pressure [42].

Due to the program’s organization in an outpatient clinic building and parental preferences, the visits took place in the afternoon (between 3 p.m. and 6 p.m.). One limitation of this study was that bioelectrical impedance analysis was not performed with the children fasting (the manufacturer recommends at least 4 h after a meal).

During the year-long program, there was an increase in both the average fat mass and muscle mass of the participating children. However, this is a physiological phenomenon during a child’s development. Therefore, changes in body fat percentage seems to be a more objective measure in children. There are no studies evaluating the normal values for fat mass or muscle mass in children. Furthermore, there are no studies determining the normal body fat percentage in the population of Polish children. The reference point in this study was the body fat norms for children developed from a German study involving over 22,000 children aged 3–16 years [22].

Initially, the number of children with an abnormally high body fat percentage was 7%. Throughout the year-long program, the proportion of children with a normal body fat percentage significantly increased from 93% to 97%. During the initial visits, a significant decrease in the average body fat percentage, a decrease of 0.5% from 23.8%, was observed, although this trend was not noted during subsequent visits.

The relatively low percentage of children with elevated body fat in the study group may be related to the fact that, in children with obesity, the BIA method may underestimate the fat mass compared to the DEXA method [45,46]. Other studies have reported that measurements obtained through BIA indicated a lower fat percentage and mass, while the fat-free mass was higher than when measured using the DEXA method [47,48]. It has been shown that the agreement between BIA and DEXA results is greater in children with severe obesity than in those with mild or moderate obesity [48].

Initially, a higher proportion of the girls (10%) had elevated body fat percentages compared to the boys (5%). However, during the year-long intervention, 98% of the girls achieved a body fat percentage within the normal range, while no significant change was observed in the boys. During the intervention, 57% of the girls and only 30% of the boys experienced a decrease in body fat percentage. The average body fat percentage decreased in the girls during the year-long program, while it increased in the boys. Therefore, the intervention resulted in a significantly better reduction in body fat percentage in the girls than in the boys. The largest number of children who achieved a decrease in fat mass was observed between the first and second visits (53% of children). During the year-long program, 17% of the children experienced a reduction in fat mass, while about 96% of the children showed an increase in muscle mass.

The average body fat percentage, fat mass, and muscle mass were higher in the children with obesity compared to the children with overweight. The change in average fat mass and muscle mass during the year-long program was independent of whether the children were in the overweight or obesity group.

Bioelectrical impedance analysis (BIA) has recently begun to be widely used in clinics, hospitals, and even commercial facilities. The widespread use of the BIA method is due to its ease of use, its non-invasiveness, and the increasing availability of this relatively inexpensive method. Importantly, body composition measurements using BIA provide reliable results [11,12,13,14]. Despite its limitations, BIA is particularly useful in monitoring changes in body composition during the treatment of excess body weight in children. Studies indicate that the greater body mass in children with obesity consists of both excess fat and fat-free mass, including bone and muscle tissue [49].

A review of meta-analyses shows a positive effect of multidisciplinary interventions on the body composition of children with excess body weight [50,51,52]. It has been shown that the desired effect was achieved especially in studies where dietary interventions were combined with physical exercise [50]. Miguel-Etayo’s meta-analysis did not show a correlation between the duration of the intervention and improvement in the body composition of children and adolescents [51]. Ho’s meta-analysis found a fat loss in study groups that was 3.2% greater than in control groups [44]. Research shows that physical exercise interventions significantly affect reductions in BMI, body weight, and body fat percentage, but their effect on changes in fat-free mass, including muscle mass, is minimal [53]. In Cai’s meta-analysis, about half of the analysed studies showed a positive effect on both fat loss and blood pressure reduction. However, nearly half of the studies showed an impact of lowering blood pressure despite having no effect on fat reduction [41].

This study showed a strong correlation between body fat percentage and anthropometric parameters such as body weight, BMI, BMI percentile, and BMI z-score. A strong correlation was also found between fat mass and anthropometric parameters such as body weight, height, BMI, BMI percentile, BMI z-score, and muscle mass. Additionally, there was a strong correlation between muscle mass and age, as well as between anthropometric parameters such as body weight, height, and BMI.

Body composition parameters and blood pressure showed only a weak positive correlation with fat mass and systolic blood pressure. This relationship was not confirmed for diastolic blood pressure. A weak correlation was observed between elevated systolic blood pressure and body weight, as well as BMI. However, no correlation was found between elevated diastolic blood pressure and the examined parameters.

A strong correlation was found between an elevated body fat percentage and an elevated BMI, confirming that the BMI index correlates well with excess fat in children with obesity and overweight. No association was found between an elevated body fat percentage and elevated blood pressure. Similar results were obtained in Bohn’s multicentre study, which showed a stronger relationship between BMI and hypertension in children than between BMI and the fat mass measured by BIA [54]. Other studies have confirmed the relationship between an elevated body fat percentage and elevated systolic and diastolic blood pressure in children who are both obese [55,56] and healthy [57]. A study by the Polish team led by Łątka demonstrated a relationship between elevated blood pressure and fat mass, fat-free mass, and BMI [58]. Differences in study results on the relationship between blood pressure and body composition parameters may be due to the relatively small sample sizes in the study groups.

Further monitoring studies are necessary to assess the percentage of children with excess body weight. Considering the proportion of children and the degree of BMI or fat tissue reduction during the one-year intervention, it is worth monitoring this group over a longer period to assess whether the results can be maintained in the following years, even after the intervention phase ends [29].

Further research is also required to evaluate the effectiveness of therapeutic programs focusing on the treatment of excess body weight in children. The goal of interdisciplinary therapies is to reduce the health risks associated with obesity. A new approach to the treatment of children who are overweight and obese emphasizes not only weight loss but also the introduction of long-term, beneficial changes that lead to a healthy lifestyle.

## 5. Conclusions

(1) During the year-long intervention program, there was a significant increase in the number of children with a normal body fat percentage, and a significant increase in muscle mass was noted in the majority of the children;
(2) Nearly 80% of the children with overweight and obesity achieved a reduction in their BMI z-score and BMI percentile;
(3) Even in such a young age group, obesity-related hypertension has been recorded;
(4) No significant impact of the year-long intervention program on the blood pressure percentiles in the children was observed;
(5) The results of the body fat percentage, fat mass, and muscle mass measurements that were obtained using the bioelectrical impedance method differed between children with overweight and obesity. The average body fat percentage, fat mass, and muscle mass were higher in the children with obesity than in the children with overweight;
(6) The best effects across all the parameters studied during the year-long program were achieved in the first three months of the project. This proves that long-term programs focused on sustained collaboration with the child and their family are necessary.

### Limitations of the Study

- The high dropout rate, with some parents withdrawing from the program before completion, remains a limitation of this study;
- BIA measurements were not performed after fasting, which may have skewed the results in case of some of the children, if they did not adhere to the recommendation of the measurement being performed at least 4 h after a meal.

## Figures and Tables

**Table 1:** Changes in BMI percentile and BMI z-score during the year-long program.

Parameter	Visit Number	N	Mean Difference ± SD	*p*
Δ BMI Percentile	1 vs. 2	391	−0.83 ± 1.49	<0.0001
	1 vs. 3	277	−1.26 ± 2.17	<0.0001
	1 vs. 4	195	−1.74 ± 3.04	<0.0001
	2 vs. 3	275	−0.49 ± 1.90	<0.0001
	3 vs. 4	194	−0.57 ± 2.48	0.0015
Δ BMI Z-score	1 vs. 2	391	−0.09 ± 0.14	<0.0001
	1 vs. 3	277	−0.13 ± 0.19	<0.0001
	1 vs. 4	195	−0.16 ± 0.21	<0.0001
	2 vs. 3	275	−0.04 ± 0.16	0.0002
	3 vs. 4	194	−0.04 ± 0.17	0.0032

**Table 2:** BMI percentile and z-score changes during the year-long program.

Parameter	Change	Visit 1 vs. 4
Δ BMI Percentile	Decrease	155 (79.49%)
	No Change	3 (1.54%)
	Increase	37 (18.97%)
N	195	
Pearson Chi^2^	*p* = 0.53	
Spearman Rank R	*p* = 0.30	
Δ BMI Z-score	Decrease	154 (78.97%)
	No Change	6 (3.08%)
	Increase	35 (17.95%)
Total	195	
Pearson Chi^2^	*p* = 0.75	
Spearman Rank R	*p* = 0.34	

**Table 3:** Changes in blood pressure percentiles during the year-long program.

Parameter	Change	Visit 1 vs. 4
Systolic BP Percentile	Decrease	78 (41.49%)
	No Change	2 (1.06%)
	Increase	108 (57.45%)
N	188	
Pearson Chi^2^	*p* = 0.11	
Spearman Rank R	*p* = 0.03	
Diastolic BP Percentile	Decrease	95 (49.48%)
	No Change	3 (1.56%)
	Increase	94 (48.96%)
N	192	
Pearson Chi^2^	*p* = 0.17	
Spearman Rank R	*p* = 0.31	

**Table 4:** Changes in mean body fat percentage by visit.

Visit Number	N	Mean Difference ± SD	*p*
1 vs. 2	383	−0.57 ± 2.33	<0.0001
1 vs. 3	268	−0.46 ± 2.99	0.0130
1 vs. 4	188	0.42 ± 3.03	0.0588

**Table 5:** Changes in body fat mass (kg).

Visit Number	N	Mean Difference ± SD	*p*
1 vs. 2	382	−0.05 ± 1.58	0.5062
1 vs. 3	267	0.48 ± 2.17	0.0004
1 vs. 4	187	1.57 ± 2.44	<0.0001

**Table 6:** Percentage of participants with a change in body fat mass between visit 1 and visit 4.

**Body Fat Mass Change**	**Visit 1 vs. 4**
Decrease	32 (17.11%)
No Change	6 (3.21%)
Increase	149 (79.68%)
Total	187
Pearson Chi^2^	*p* = 0.0002
Spearman Rank R	*p* < 0.0001

**Table 7:** Changes in muscle mass (kg).

Visit Number	N	Mean Difference ± SD	*p*
1 vs. 2	384	0.61 ± 1.94	<0.0001
1 vs. 3	268	1.44 ± 2.03	<0.0001
1 vs. 4	189	3.40 ± 2.41	<0.0001

**Table 8:** Percentage of participants with a change in muscle mass between visit 1 and visit 4.

Muscle Mass Change	Visit 1 vs. 4
Decrease	7 (3.70%)
No Change	0 (0.00%)
Increase	182 (96.30%)
Total	189
Pearson Chi^2^	*p* < 0.0001
Spearman Rank R	*p* < 0.0001

**Table 9:** BMI in boys and girls.

BMI Percentile	Girls		Boys	
	Visit 1	Visit 4	Visit 1	Visit 4
Normal (<85th)	0%	4.6%	0%	0%
Overweight (≥85–95th)	37.4%	54.6%	7.6%	19.5%
Obesity (≥95th)	62.6%	40.8%	92.4%	80.5%

**Table 10:** Blood pressure changes in boys and girls.

BP Percentile		Girls		Boys	
		Visit 1	Visit 4	Visit 1	Visit 4
Systolic BP	Normal (<90c)	91.54%	96.15%	88.36%	81.61%
	High Normal	4.04%	2.88%	5.17%	8.05%
	Hypertension	4.41%	0.96%	6.47%	10.34%
Pearson Chi^2^		*p* = 0.0536		*p* = 0.0759	
Spearman Rank R		*p* = 0.0528		*p* = 0.3315	
Diastolic BP	Normal (<90c)	67.87%	72.38%	62.25%	73.56%
	High Normal	11.19%	12.38%	16.95%	11.49%
	Hypertension	20.94%	15.24%	17.80%	14.94%
Pearson Chi^2^		*p* = 0.1413		*p* = 0.1401	
Spearman Rank R		*p* = 0.0503		*p* = 0.0373	

**Table 11:** Body fat percentage in girls and boys.

Body Fat Percentage	Girls		Boys	
	Visit 1	Visit 4	Visit 1	Visit 4
Within Norm (<90c)	90.37%	98.15%	95.28%	95.40%
Excess (>90c)	9.63%	1.85%	4.72%	4.60%
Pearson Chi^2^	*p* = 0.0097		*p* = 0.5307	
Spearman Rank R	*p* = 0.0070		*p* = 0.7896	

**Table 12:** Blood pressure in children who were overweight and obese.

Parameter	Visit Number	Overweight	Obese	*p*
Systolic BP Percentile	1	40.56 ± 24.79	52.52 ± 28.58	<0.0001
	2	39.48 ± 26.79	49.32 ± 27.78	0.0027
	3	37.77 ± 26.10	48.50 ± 28.46	0.0073
	4	39.67 ± 25.43	52.31 ± 28.71	0.0106
Diastolic BP Percentile	1	71.43 ± 22.87	76.09 ± 21.19	0.0386
	2	64.81 ± 25.57	72.66 ± 23.46	0.0058
	3	63.11 ± 26.71	70.44 ± 24.00	0.0375
	4	68.36 ± 25.74	72.48 ± 22.66	0.3133

**Table 13:** Mean body fat percentage in children who were overweight or obese (by gender).

Gender	Visit Number	Overweight	Obese	*p*
Girls	1	24.61 ± 3.04	27.80 ± 2.72	<0.0001
Boys	1	16.02 ± 1.83	20.93 ± 4.51	<0.0001
Girls	2	24.72 ± 2.38	26.55 ± 3.04	<0.0001
Boys	2	15.53 ± 2.82	20.60 ± 4.99	0.0001
Girls	3	24.63 ± 2.87	26.02 ± 3.53	0.0477
Boys	3	15.58 ± 2.75	20.98 ± 5.49	0.0010
Girls	4	24.96 ± 2.51	26.15 ± 3.14	0.0477
Boys	4	17.54 ± 4.94	22.16 ± 4.71	0.0368

**Table 14:** Mean body fat mass (kg) in children who are overweight or obese.

Gender	Visit Number	Overweight	Obese	*p*
Girls	1	9.44 ± 1.49	12.12 ± 2.84	<0.0001
Boys	1	6.16 ± 1.31	9.73 ± 3.72	0.0001
Girls	2	9.55 ± 1.43	11.50 ± 2.92	<0.0001
Boys	2	6.18 ± 1.80	10.00 ± 4.19	0.0004
Girls	3	10.07 ± 1.92	11.83 ± 3.30	0.0004
Boys	3	6.48 ± 1.97	10.61 ± 4.84	0.0040
Girls	4	10.90 ± 2.05	12.43 ± 3.02	0.0062
Boys	4	7.94 ± 3.27	11.67 ± 5.18	0.1166

**Table 15:** Mean muscle mass in children who are overweight or obese (kg).

Parameter	Visit Number	Overweight	Obese	*p*
Muscle Mass	1	26.80 ± 4.08	31.01 ± 4.60	<0.0001
	2	27.55 ± 3.96	31.74 ± 4.81	<0.0001
	3	29.12 ± 4.08	32.49 ± 4.89	<0.0001
	4	30.46 ± 3.93	34.09 ± 4.92	<0.0001

**Table 16:** Pearson correlations between body composition, anthropometric parameters, and blood pressure in girls and boys.

x	Y	Girls			Boys		
		N	r (X,Y)	*p*	N	r (X,Y)	*p*
Body Fat Percentage	Body weight	729	0.41	<0.0001	635	0.79	<0.0001
	Z-score body weight	729	0.40	<0.0001	635	0.66	<0.0001
	BMI percentile	729	0.58	<0.0001	635	0.70	<0.0001
	BMI	729	0.71	<0.0001	635	0.91	<0.0001
	Z-score BMI	729	0.66	<0.0001	635	0.80	<0.0001
	Systolic BP	722	0.12	0.0008	631	0.35	<0.0001
	Diastolic BP	722	0.16	<0.0001	631	0.42	<0.0001
	Systolic BP percentile	714	0.09	0.0139	627	0.34	<0.0001
	Diastolic BP percentile	721	0.13	0.0006	630	0.33	<0.0001
Body Fat Mass	Body height	729	0.56	<0.0001	635	0.52	<0.0001
	Body weight	728	0.87	<0.0001	635	0.91	<0.0001
	Z-score body weight	728	0.64	<0.0001	635	0.70	<0.0001
	BMI percentile	728	0.50	<0.0001	635	0.60	<0.0001
	BMI	728	0.89	<0.0001	635	0.91	<0.0001
	Z-score BMI	728	0.63	<0.0001	635	0.72	<0.0001
	Systolic BP	721	0.31	<0.0001	631	0.42	<0.0001
	Diastolic BP	721	0.24	<0.0001	631	0.43	<0.0001
	Systolic BP Percentile	713	0.25	<0.0001	627	0.40	<0.0001
	Diastolic BP Percentile	720	0.19	<0.0001	630	0.30	<0.0001
Muscle Mass	Body height	729	0.91	<0.0001	636	0.87	<0.0001
	Z-score body height	729	0.73	<0.0001	636	0.64	<0.0001
	Body weight	728	0.94	<0.0001	636	0.91	<0.0001
	Z-score body weight	728	0.62	<0.0001	636	0.66	<0.0001
	BMI	728	0.62	<0.0001	636	0.63	<0.0001
	Systolic BP	721	0.39	<0.0001	632	0.44	<0.0001
	Diastolic BP	721	0.20	<0.0001	632	0.28	<0.0001
	Systolic BP Percetile	713	0.32	<0.0001	628	0.39	<0.0001
	Diastolic BP Percentile	720	0.17	<0.0001	631	0.16	<0.0001

**Table 17:** Pearson correlations between the systolic blood pressure percentile and body composition.

Variable	Visit	Normal BP (<90c)			High Normal BP (≥90–95c)			Hypertension (≥95c)			*p*	R
		N	Mean	SD	N	mean	SD	N	Mean	SD		
Body weight	1	454	42.04	6.80	23	48.85	9.05	27	52.16	7.02	<0.0001	0.36
	4	179	47.25	8.22	7	52.16	9.12	6	55.00	8.66	0.0286	0.19
Z-score body weight	1	454	1.95	0.45	23	2.28	0.47	27	2.53	0.40	<0.0001	0.30
	4	179	1.81	0.50	7	2.04	0.38	6	2.20	0.48	0.0897	0.16
BMI	1	454	22.41	2.13	23	24.08	2.69	27	25.58	3.23	<0.0001	0.33
	4	179	22.79	2.44	7	23.60	2.21	6	25.47	4.23	0.0289	0.19
Body fat mass	1	442	10.03	3.06	23	12.64	4.15	27	14.15	4.06	<0.0001	0.32
	4	179	11.53	3.93	7	13.46	4.67	6	13.25	5.60	0.2833	0.12
Body muscle mass	1	444	29.62	4.62	23	33.40	5.33	27	35.02	3.85	<0.0001	0.30
	4	179	33.01	4.86	7	35.74	5.60	6	38.58	3.62	0.0097	0.22

## Data Availability

The data presented in this study are available on request from the corresponding author. The data are not publicly available due to ethical restrictions.

## Supplementary Materials

The following supporting information can be downloaded at: https://www.mdpi.com/article/10.3390/jcm14103489/s1, Nutritional recommendation.

## Abbreviations

BP	blood pressure
BMI	body mass index
BIA	bioelectrical impedance analysis
DEXA	Dual-Energy X-ray Absorptiometry
SD	standard deviation
OLAF	name of a nationwide, nationally representative epidemiological study PL0080 aimed at assessing anthropometric parameters
