# Supplementary material for: Changes in Anthropometric Measurements, Body Composition, and Blood Pressure in 6–10-Year-Old Children with Overweight and Obesity in Szczecin During a Year-Long Intervention Program
Source: J Clin Med. 2025 May 16;14(10):3489. doi: 10.3390/jcm14103489 (PMC12111922; doi:10.3390/jcm14103489)
Supplement: Supplementary file 1 [file jcm-14-03489-s001.zip › jcm-3534831-supplementary.pdf]

Nutritional Recommendations
Source: "The Brave Eight" Program

1. **Prepare and eat a properly balanced breakfast every day before going to school.** On weekends or non-school days, remember to have breakfast within the first hour after waking up. Include unrefined products in your breakfast for more energy, as well as natural dairy products and your first portion of fruit or vegetables.
2. **Take a packed second breakfast from home.** Remember to eat it about 3 hours after your first meal at home. Wholegrain sandwiches with plenty of your favorite vegetables and a source of protein (such as cottage cheese, lean ham, or hard cheese) are a great option—don't forget a bottle of water. If you stay at school for a long time, take an extra portion of fruit. On non-school days, explore the flavors of fruits and vegetables in colorful salads.
3. **Eating 4–5 regular meals a day with appropriate composition and volume is essential for maintaining stable blood sugar levels.** Decide which of the planned meals will be your main lunch. Avoid eating two meals of similar size during the day.
4. **Make sure there's about a 3-hour break between meals.** If lunch is eaten at school, plan a small meal when you get home. If lunch is eaten at home, take at least two meals to school (a second breakfast and, for example, a portion of vegetables, seeds, natural yogurt, or fruit).
5. **Eat a light dinner at least 2 hours before bedtime.** Dinner should be smaller than breakfast.
6. **When planning meals, remember that nutritional and energy value is influenced by factors such as cooking method and additives.** Limit sauces, mayonnaise, cream, and roux. Cook, stew, and bake more often. Keep in mind that breading increases the caloric value and lowers the nutritional value of a dish.
7. **Avoid snacking between meals.** Maintain at least a 3-hour interval between meals. Drink water.
8. **When preparing meals, include products that are as unrefined as possible.** Wholegrain bread, homemade muesli, coarse groats, or wholegrain pasta are excellent choices.
9. **Include raw or lightly steamed vegetables in your meals.** Eat colorful foods.
10. **Once or twice a day, add your favorite portion of fruits or vegetables to a meal.** A portion may be one large fruit (e.g., an apple), three small ones (e.g., mandarins), or a glass of small fruits (e.g., berries).
11. **Confidently include a portion of seeds such as nuts, pumpkin seeds, or sunflower seeds in your meals.** During the week, legumes can replace one main meal.
12. **At least once a week, include a portion of fish with lunch.** Be adventurous in exploring the bounty of the seas and lakes. When including fish in your lunch, ensure high nutritional value—avoid breading and deep-frying. Bake, stew, or steam instead.
13. **Choose natural products more often.** Confidently combine them with portions of fruits and vegetables. Limit the intake of processed foods with sweet additives. Read labels carefully. Choose dairy products with controlled fat content.
14. **Limit sweets and eliminate sugary drinks.**
15. **No other drink can replace water.** Water should be consumed throughout the day between meals.
16. **Limit your intake of fast food.** It has low nutritional value.
17. **Shared meals are a great time to talk.** Eating together helps explore new flavors—sometimes inspiration for a new taste comes unexpectedly, so it's worth making time for it.
18. **Reinforce new eating habits.**
